# Draft genome sequence of *Nocardioides* strain MAHUQ-72 isolated from soil sample of a rice field located in Anseong, South Korea

**DOI:** 10.1128/mra.01066-25

**Published:** 2025-11-25

**Authors:** Md. Amdadul Huq, Shahina Akter, Md. Shahidul Islam, Md. Shahedur Rahman

**Affiliations:** 1 Department of Life Sciences, College of BioNano Technology, Gachon University 65440, Seongnam, Republic of Korea; 2 Department of Food Science and Biotechnology, Gachon University 65440, Seongnam, Republic of Korea; 3 Department of Microbiology, Faculty of Science and Engineering, Rabindra Maitree University, Kushtia, Bangladesh; 4 Department of Genetic Engineering and Biotechnology, Jashore University of Science and Technology 421984, Jashore, Bangladesh; Portland State University, Portland, Oregon, USA

**Keywords:** genome sequence, *Nocardioides*strain MAHUQ-72, rice field

## Abstract

This study reports the draft genome sequence of *Nocardioides* strain MAHUQ-72, a bacterial strain isolated from the soil sample of a rice field located in Anseong, South Korea, in 2020. The genome consists of 4,732,459 base pairs assembled into 23 contigs, encoding 4,555 predicted protein-coding genes.

## ANNOUNCEMENT

The genus *Nocardioides* of the family *Nocardioidaceae* belongs to the phylum *Actinomycetota* and was first described by Prauser (1), currently comprises 171 validly published species (2) that have been isolated from different environments, including soil, water, plant roots, oil shale columns, meadows, deserts, forests, dandelions, sewage sludge, and caves (3). During investigations on bacterial biodiversity in a rice field, a new strain of the genus *Nocardioides,* designated *Nocardioides* strain MAHUQ-72, was isolated, and its draft genome assembly is presented in this study.

Strain MAHUQ-72 was isolated from the soil sample of a rice field (Date of collection: 2020.10.20), located in Anseong, South Korea (37° 00′ 31″ N, 127° 21′ 58″ E). One gram of soil sample was suspended in 9 mL of sterile 0.85% (wt/vol) NaCl solution. The suspension was serially diluted up to a 10^−6^ dilution, and 100 µL of individual dilution was spread onto R2A agar plates (4). Then, the plates were placed in a 30°C incubator for 3 days. Single colonies were purified by repeated streaking on fresh R2A agar plates. The strain MAHUQ-72 has been deposited to the China General Microbiological Culture Collection Center with deposition number CGMCC 1.19066. The genomic DNA was extracted from the bacterial culture that was grown in R2A broth for 24 husing Solg Genomic DNA Prep kit (Solgent, Republic of Korea), according to the manufacturer’s protocol. The DNA fragments were size selected using AMPure XP beads to isolate those within the desired range, typically 200–300 base pairs (bp). The library for sequencing was prepared using the Nextera XT DNA Library Prep Kit (Illumina, San Diego, USA). After library preparation, sequencing was carried out using the Illumina HiSeq 3000 platform with paired-end 2×150 bp reads following the standard protocol provided by the manufacturer. Illumina’s bcl2fastq software version 2.20.0 was utilized with default parameters to demultiplex the raw reads and convert them into FASTQ format. Reads were processed with Trimmomatic version 0.38 (5). The genome sequence was assembled by SOAPdenovo v2.04 assembler (6). The genome was annotated with the NCBI Prokaryotic Genome Annotation Pipeline version 6.3 (7–9). The quality analysis was performed by CheckM, version 1.2.4 (10). The strain was identified using a whole genome-based taxonomic analysis in the Type (Strain) Genome Server (https://tygs.dsmz.de). Default parameters were used for all software unless otherwise specified. The genome-to-genome distance bioinformatics tool (11) was used to measure *in silico* DNA-DNA hybridization (isDDH) values. To estimate the degree of pairwise relatedness between MAHUQ-72 and the closest type strain, BLAST-based average nucleotide identity (ANI) was calculated (12). The best matches to the strain MAHUQ-72 genome are the type strain *Nocardioides koreensis* JCM 16022 (accession GCA_039531775.1)with isDDH and ANI values of 27.6 and 84.2%, respectively.

The genome of *Nocardioides* strain MAHUQ-72 is 4,732,459 bp long with a GC content of 72.5%. The assembly contains 23 contigs with a coverage of 83×. A total of 4,620 genes were predicted, of which 4,555 are protein-coding genes. The details of genomic features are presented in Table 1.

**TABLE 1:** Genomic features of *Nocardioides* strain MAHUQ-72

Parameter	Result
Description of source	
Location	Anseong, South Korea
Time	2020
Type	Soil sample of a rice field
Sequencing summary	
Total genome length	4,732,459 bp
Coverage	83×
GC content	72.5%
Number of reads	2,982,010
Assembly report	
Contigs	23
Contig *L*_50_	5
Contig *N*_50_	453.6 kb
Genome length	4.7 Mb
Annotation report	
Genes (total)	4,620
CDSs (total)	4,566
CDSs (with protein)	4,555
ncRNAs	3
tRNA	48
Quality analysis	
Completeness	99.47%
Contamination	1.67%

## Data Availability

The draft genome sequence of *Nocardioides* strain MAHUQ-72 was deposited in GenBank under the accession number JBQWTR000000000. The raw reads are available in SRA under the accession number SRR33604977.The version described in this paper is the first version, JBQWTR000000000.1.
